# Chemical Structure and Microstructure Characterization of Ladder-Like Silsesquioxanes Derived Porous Silicon Oxycarbide Materials

**DOI:** 10.3390/ma14061340

**Published:** 2021-03-10

**Authors:** Jakub Marchewka, Piotr Jeleń, Izabela Rutkowska, Patryk Bezkosty, Maciej Sitarz

**Affiliations:** Faculty of Material Science and Ceramics, AGH University of Science and Technology, al. Mickiewicza 30, 30-059 Kraków, Poland; pjelen@agh.edu.pl (P.J.); irutkowska@agh.edu.pl (I.R.); bezkosty@agh.edu.pl (P.B.); msitarz@agh.edu.pl (M.S.)

**Keywords:** silsesquioxanes, silicon oxycarbide, porous ceramics, sol-gel synthesis

## Abstract

The aim of this work was to synthesize porous ceramic materials from the SiOC system by the sol-gel method and the subsequent pyrolysis. The usage of two types of precursors (siloxanes) was determined by Si/C ratio in starting materials. It allows us to control the size of the pores and specific surface area, which are crucial for the potential applications of the final product after thermal processing. Methyltrimethoxysilane and dimethyldiethoxysilane were mixed in three different molar ratios: 4:1, 2:1, and 1:1 to emphasize Si/C ratio impact on silicon oxycarbide glasses properties. Structure and microstructure were examined both for xerogels and obtained silicon oxycarbide materials. Brunauer-Emmett-Teller (BET) analysis was performed to confirm that obtained materials are porous and Si/C ratio in siloxanes precursors affects porosity and specific surface area. This kind of porous ceramics could be potentially applied as gas sensors in high temperatures, catalyst supports, filters, adsorbents, or advanced drug delivery systems.

## 1. Introduction

Porous ceramic materials have been successfully used in many industries, including thermal insulation materials [1,2], filters [3], membranes [4], adsorbents [5], catalyst supports [6], and materials for medicine [7]. The huge variety of methods of their preparation allows us to control the most important properties, such as permeability, specific surface area, shape, and size of the pores in a wide range. One of the traditional and simplest methods of obtaining porous ceramics is sintering of ceramic powders. Generally this method is used to receive materials with low porosity in the order of 30% [8]. More sophisticated and modern methods, such as additive manufacturing and other rapid prototyping techniques, enable to obtain materials with precisely defined porosity and designed shape [9,10].

In an unconventional way, porous ceramics can also be received from preceramic polymers as starting precursors. This type of materials is classified as a Polymer Derived Ceramics (PDCs). The first mention of them comes from the 1960s, when Ainger and Herbert [11], and Chantrell and Popper [12], submitted the receiving of nonoxide ceramics from molecular precursors. Nowadays, the most known and investigated classes of PDCs are Si_3_N_4_, SiC, SiCN, SiOC, and also a quaternary system with the addition of boron or aluminum to SiCN and SiOC systems [13]. Among them, silicon oxycarbide glass (from SiOC system) seems to be particularly interesting due to its high chemical and thermal resistance [14,15] and ability to introduce porosity in a relatively easy way [16].

Silicon oxycarbide (SiOC) glasses are materials with an amorphous silica structure, in which two oxygen anions O^2−^ are replaced by one carbon anion C^4−^. Such anionic substitution in silica glass network leads to a local increase in the density of bonds, so that mechanical strength and thermal stability (up to 1500 °C) are significantly improved [17,18,19]. Theoretically, all the basic properties of SiOC glasses will be ameliorated as the amount of carbon incorporated into the glass structure increases. However, silica glass structure can accept only a limited amount of carbon anions and the rest occur as a free carbon phase in practice [20]. The consequence is deterioration of some physicochemical properties and black color of the received materials (hence the SiOC glasses are interchangeably known as black glasses) [21,22]. Researches on the black glasses were generally focused on solid materials or coatings for high-temperature applications [23,24], bioactive components [25,26], and some electronic applications (e.g., microelectromechanical systems) [27]. Nowadays, porous SiOC glasses are gaining increasingly importance because of their possible applications as anode material in Li-ion intercalation batteries [28], catalyst supports [29], sorbents [30], gas sensors [31], and drug delivery systems [32]. Worth mentioning is fact that such materials are characterized with properties comparable to aluminum oxide, like high porosity, which is related to high surface area, chemical inertness in aggressive environments, and thermal stability. One of the main innovative applications of Al_2_O_3_ is in the field of catalysis as catalyst support, therefore SiOC materials may be a perfect alternative in this application [33,34].

Silicon oxycarbide materials have been received over the decades by numerous methods: Chemical vapor deposition (CVD) and its modifications [35,36], magnetron sputtering [37], spark plasma sintering (SPS) [38], and the others [13]. These methods are quite complicated and also require expensive laboratory equipment. On the other hand, a simple and relatively effective route to obtain black glasses is to use the sol-gel method with appropriate polymeric precursors which contain Si-C bonds [39,40]. These bonds are responsible for durability both of xerogels and glass, which is obtained after thermal processing [41]. It is during the pyrolysis process when the pores are formed, and their size and shape depend mainly on precursors and the type of solvent used in synthesis [42,43,44].

The aim of this work was to obtain silicon oxycarbide materials with a high surface area. The amorphous materials from SiOC system were received from ladder-like polysilsesquioxanes. Moreover, the study evaluated the impact of Si/C ratio of starting precursors as the key properties for porous ceramics. In addition, the potential applications for these materials, such as high-temperature gas sensors or highly efficient adsorbents and catalyst supports, were also indicated. 

## 2. Materials and Methods

Preparation of polysiloxane sol—Methyltriethoxysilane (MTES, ≥98%, Sigma-Aldrich, Saint Louis, MO, USA) and dimethyldiethoxysilane (DMDES, ≥97%, Sigma-Aldrich) were used as the substrates for sol-gel synthesis of ladder-like polysilsesquioxanes according to our procedure described previously [38]. It is known [45] that during the synthesis of ladder-like polysilsesquioxanes, MTES and DMDES allow the introduction of T (three oxide ions and one carbon ion by one silicon ion) and D (two oxide ions and two carbon ion by one silicon ion) structural units, respectively, into the final product. Briefly, both reagents were mixed in 1:1, 2:1, and 4:1 volume ratio using ethanol (99.8%, Avantor Performance Materials, Gliwice, Poland) as a solvent. Then, the mixture was hydrolyzed with diluted (pH = 4.5) 1M hydrochloric acid (Avantor Performance Materials). After subsequent polycondensation, three types of sol solutions (T:D 1:1, T:D 2:1, and T:D 4:1, respectively) were obtained.

Preparation of silicon oxycarbide materials—T:D 1:1, T:D 2:1, and T:D 4:1 sols were poured into Petri dishes and dried at 70 °C for 7 days. Obtained xerogels were described as S1, S2, and S3 samples, respectively. Then, they were pyrolyzed in a nitrogen atmosphere (99.999%, Air Liquide, Kraków, Poland) using the procedure including the following stages—heating at the rate of 5 °C/min, holding at 800 °C for 30 min, and subsequent cooling to the room temperature inside the furnace. The inert gas was applied to avoid the oxidation of the material and the loss of carbon from the product structure. Samples S1–S3 after the pyrolysis were described as S4–S6, respectively. Samples were obtained as loose bulk materials.

X-Ray Diffraction (XRD)—X-Ray diffractograms of S1–S6 samples were obtained with X’Pert Pro (Malvern Panalytical, Malvern, UK) diffractometer. CuKα1 radiation was applied in Bragg-Brentano geometry in the 2θ range from 10 to 90° and at the step of 0.008° using Debye-Scherrer-Hull (DSH) method.

Fourier-transformed Infrared spectroscopy (FT-IR)—the structure of S1–S6 samples was analyzed by FT-IR spectroscopy using Vertex 70v (Bruker, Billerica, MA, USA) spectrometer and standard KBr pellet technique. The measurements in the absorbance mode were performed in the range from 400 to 4000 cm^−1^, at the resolution of 4 cm^−1^, and collecting 128 scans.

Magic Angle Spinning Nuclear Magnetic Resonance (MAS NMR)—MAS-NMR spectra for S2 and S4 samples were recorded on Avance 500 MHz (Bruker) spectrometer with a probe size of 4 mm. The parameters of the analysis were as follows: Spinning speed of 15 kHz, 3000 scans with a total accumulation time of 100 min for ^13^C MAS NMR and spinning speed of 5 kHz, and 4500 scans with a total accumulation time of 150 min for ^29^Si MAS NMR. Spectral deconvolution was conducted based on Levenberg-Marquardt algorithm using Bruker OPUS 7.2 software [46].

Brunauer-Emmett-Teller (BET) surface area analysis—the specific surface area and the pore size distribution of S4–S6 samples were evaluated based on BET adsorption isotherms for nitrogen using ASAP 2010 system (Micromeritics Instrument Corporation, Norcross, GA, USA). The samples were degassed at 300 °C for 72 h before the measurements.

## 3. Results and Discussion

The basic structural units of silsesquioxane ladders are T units [47]. On the other hand, D units most often form oligo- or polysilsesquioxanes [47]. Their application in a specific molar ratio and with the appropriate conditions of the polycondensation reaction makes it possible to strictly define their structure. Therefore, after their subsequent pyrolysis silicon oxycarbide materials with a controlled amount of Si-C bonds could be obtained. Depending on the conditions of the thermal processing during the whole procedure (drying, pyrolysis) the porous microstructure of the final product is generated. In this work, these parameters were determined based on our previous results [20,40,48]. However, to the best of our knowledge, porous SiOC materials obtained from ladder-like silsesquioxanes have not yet been investigated. Therefore, it was decided to mix both reagents in three different proportions: 1:1, 2:1, and 4:1, to examine the influence of Si/C ratio in starting materials on structural and microstructural characterization and most important properties for final porous products.

**Figure 1:**
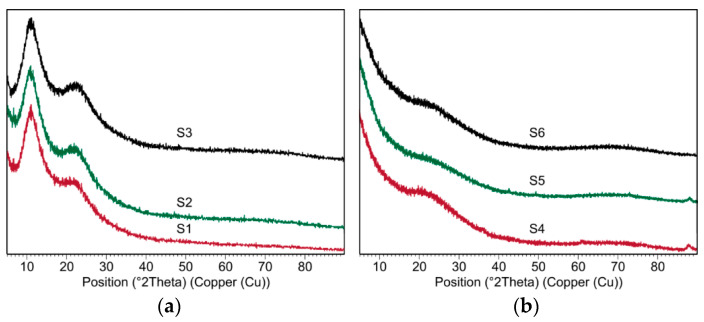
XRD diffraction patterns of (**a**) the xerogels (S1–S3 samples) and (**b**) the silicon oxycarbide materials (S4–S6 samples).

X-Ray diffraction examination was conducted for all samples, i.e., the xerogels and the silicon oxycarbide materials (Figure 1). For the S1–S3 samples before thermal processing, there are no characteristic crystalline peaks observed, therefore their structure could be considered as amorphous. Additionally, two regions of background rise, the so called amorphous “halo”, are observed in 2Theta ranges 8–12° and 20–28°. Such an observation is typical for ladder like-structures. The first reflection is due to the limited degree of order of the intermolecular chain distance, while the second one can be the result of the distance between the ladder structure [45,48]. Samples after thermal processing at 800 °C similarly do not show any crystalline peaks, which classify their structure as amorphous. There is one broad amorphous “halo” at range 18–24° 2Theta, which is characteristic for SiOC materials.

**Figure 2:**
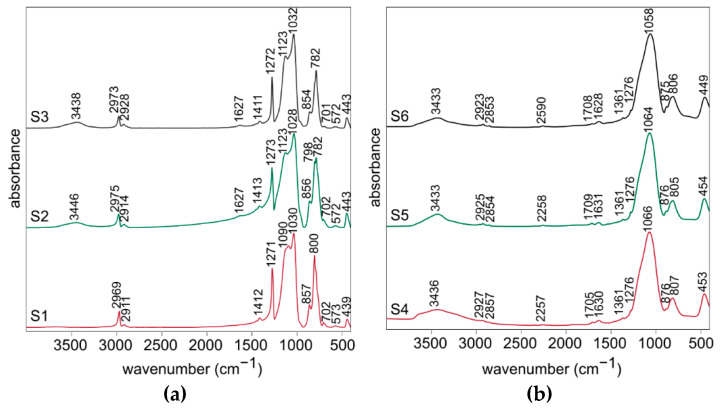
FT-IR spectra for (**a**) the xerogels (S1–S3 samples) and (**b**) the silicon oxycarbide materials (S4–S6 samples).

The FT-IR spectra of all xerogels (samples S1–S3) are very similar (Figure 2a). The two most intense bands observed at about 1030 cm^−1^ and around 1090–1120 cm^−1^ are specific for polysilsesquioxanes ladder-like structure and they are attributed to the stretching vibrations of Si-O-Si bridges and rings, respectively [20,49]. Furthermore, the weak band at about 570 cm^−1^ could be assigned to the stretching vibrations of four-fold Si-O-Si rings typical for ladder-like structures and the noticeable one at about 440 cm^−1^ to the Si-O bending vibration [20,40,50]. Numbers of bands visible in the spectra are associated with the presence of Si-CH_3_ groups in the obtained polysilsesquioxanes. These visible between 700 and 800 cm^−1^ are responsible for the stretching vibrations of Si-C bonds, whereas the ones at about 1270 cm^−1^ and 1410 cm^−1^ for the stretching vibrations and at about 850 cm^−1^ for the bending vibrations of C-H bonds. The two bands at about 2920 cm^−1^ and 2970 cm^−1^ are typical for the stretching vibrations of C-H bonds [50,51]. The broad band at about 3440 cm^−1^ and the weak one at 1627 cm^−1^ are attributed to the stretching and bending modes of O-H bond in water. This indicates the presence of water molecules absorbed in S2 and S3 samples and could be associated with their higher porosity compared to S1 sample. 

The FT-IR spectra of all obtained silicon oxycarbide materials (samples S4–S6) also closely resemble each other, but they are much different compared to the ones for the xerogels (Figure 2b). This shows that the significant changes of the structure occurred during the pyrolysis. The most intense band at about 1060 cm^−1^ is attributed to the stretching vibrations in Si-O-Si structure. Then, the next significant ones, visible at about 805 cm^−1^ and 450 cm^−1^, are assigned to the Si-O-Si stretching and O-Si-O bending vibrations. The bands at about 2260 cm^−1^ for the stretching vibration and about 875 cm^−1^ for the bending vibration could be assigned to the reactive Si-H bond [49,52]. Their presence may be beneficial in case of the potential applications of the materials in catalysis. Moreover, the very weak band at about 1710 cm^−1^ could be explained as the stretching vibration of C=O bond in the small share of oxidized free carbon phase [20]. A few very weak bands at about 2925 cm^−1^, 2855 cm^−1^, as well as at 1361 cm^−1^ and 1276 cm^−1^ are associated with the stretching vibrations of C-H bonds in the polysilsesquioxanes. This indicates the presence of the organic phase remnants in the material after the pyrolysis. It is possible that they will be removed with the application of the higher temperature in the process. As it was mentioned before, 3440 cm^−1^ and 1627 cm^−1^ bands are specific for O-H bond in water. They are more intense compared to these observed in the spectra for xerogels. This may suggest that the porosity of the material after the pyrolysis is significantly higher.

Detailed FT-IR spectra analysis for the xerogels and silicon oxycarbide materials are presented in Table 1 and Table 2.

**Figure 3:**
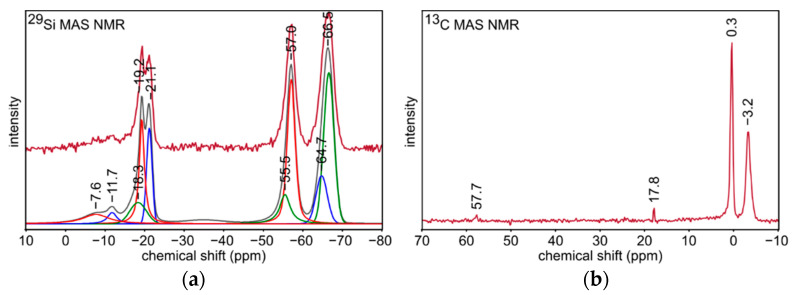
(**a**) ^29^Si and (**b**) ^13^C MAS NMR spectra for S2 sample.

^29^Si and ^13^C MAS NMR experiments were performed in one series of the samples based on T:D 2:1 polysilsesquioxanes sol, i.e., S2 xerogel and S4 silicon oxycarbide material. Spectral deconvolution was performed to identify the individual signals. Nine of them grouped in two ranges between 0 and −25 ppm as well as between −50 and −70 ppm were distinguished in ^29^Si MAS NMR spectrum for S2 sample (Figure 3a). They were related to silicon atoms in D and T structural units, respectively. The evidence of ladder-like polysilsesquioxane structure preparation is given by the signals at −64.7 and −66.5 ppm [50,53,54]. The presence of -OSi(CH_3_)_2_- units in the xerogel structure is proven by the signal at −21.1 ppm, whereas terminal -OSi(CH_3_)_2_OH groups are assigned to the signal at −11.7 ppm [50,55]. Moreover, the signals at −7.6, −18.3, and −19.2 ppm may be associated with trimeric and tetrameric polycyclosiloxanes derived from DMDES molecules which did not generate ladder-like structure [50,55]. Two signals at −55.5 and −57.0 ppm derived from open-chain oligomers. ^13^C MAS NMR spectrum for S2 sample (Figure 3b) was collected to get a complementary information about the xerogel structure. Intensive signals at −3.2 and 0.3 ppm are related to carbon atoms in Si-CH_3_ groups of D and T structural units, respectively, which confirms the preservation of Si-CH bonds in the xerogel [50,56]. Besides, weak signals at 17.8 and 57.7 ppm could be assigned to -SiOEt groups in the remaining unreacted molecules of the substrates, most probably D units, as seen in Figure 3a ^29^Si NMR [57].

**Figure 4:**
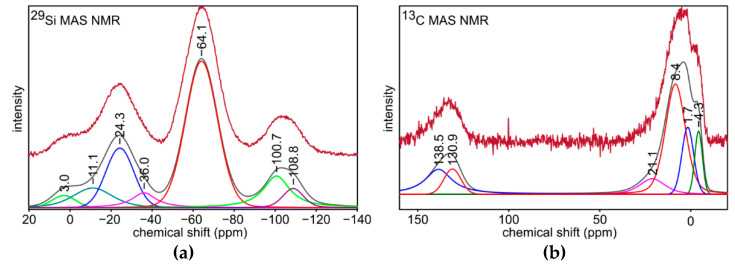
(**a**) ^29^Si and (**b**) ^13^C MAS NMR spectra for S4 sample.

^29^Si MAS NMR spectrum for S4 sample (Figure 4a) indicates its complex structure. The most intense signal at −64.1 ppm is assigned to silicon atoms in [SiO_3_C] (T structural units). Other signals could be associated as follows: 3.0 ppm to [SiOC_3_] (M structural units), −36.0 ppm to [SiO_2_C_2_] (D), −100.7 ppm to [SiO_3_OH] (Q^3^), −108.8 ppm to [SiO_4_] (Q^4^), and −11.1 ppm to [SiC_4_] (X) [54,57]. They are typical to the structure of silicon oxycarbide, and therefore this confirms the preparation of this material during the pyrolysis. Furthermore, the signal at −24.3 ppm may be related to [HSiO_3_] (D^H^ structural units) [58]. This is consistent with FT-IR analysis, which shows the presence of Si-H bonds in the material. Based on the integral intensities obtained during the mathematical deconvolution of the ^29^Si NMR spectrum, the percentages of individual structural units were calculated (Table 3). The analysis shows that [SiO_3_C] (T structural units) is the dominant phase, followed by [SiO_2_C_2_] (D) phase. Additionally, the ratio of T units to D units equals 2.2:1, which is almost the starting value/desired value (2:1). Additional information is given by ^13^C MAS NMR spectrum for S4 sample (Figure 4b). Signals between 25 and −5 ppm are typical for carbon bonded to silicon atoms in -Si-CH_x_ groups [59]. Therefore, a small amount of them derived from T and D structural units remains in the material after the pyrolysis. These signals may be associated this way: −4.3 and 1.7 ppm to -Si-CH_3_ groups from T and D structural units and 8.4 ppm to -Si_3_-CH groups [40]. Furthermore, the signal at 21.1 ppm is typical for [SiC_4_] structural units [59]. Other signals at 138.5 and 130.9 ppm may be explained as related to C=O bonds in the small share of oxidized free carbon phase, which is also confirmed by FT-IR analysis [60]. The results of ^13^C MAS NMR experiment are consistent with ^29^Si MAS NMR, and they also confirm the preparation of silicon oxycarbide during the pyrolysis. 

Structural analysis indicates that sol-gel synthesis let to prepare ladder-like polysilsesquioxanes build of methyl- and hydroxyl-terminated T and D structural units. A small amount of the remaining ethoxy groups shows that the polycondensation reaction was not fully completed. The presence of -Si-CH_3_ groups in the xerogel structure is crucial for the preparation of silicon oxycarbide. During the pyrolysis Si-C bonds are preserved and therefore carbon atoms are introduced in the silicon oxide structure.

**Figure 5:**
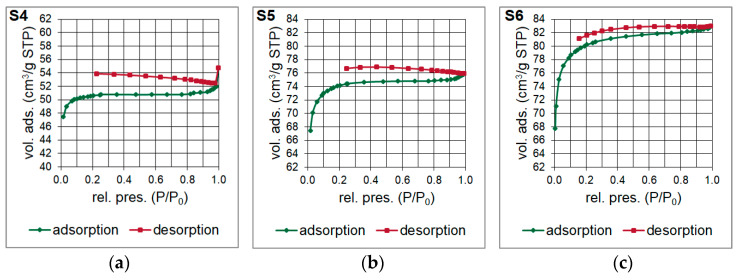
(**a**–**c**) Nitrogen adsorption-desorption isotherms of the silicon oxycarbide materials (S4–S6 samples), abbreviations: vol. ads.—volume adsorbed, rel. pres.—relative pressure.

Brunauer-Emmett-Teller (BET) surface area analysis was performed to examine the specific surface area and the pore size distribution of the silicon oxycarbide materials. Two types of nitrogen adsorption-desorption isotherms were obtained for the samples (Figure 5). Type II isotherm was observed for S4 sample, which suggest the multilayer formation during the analysis, and therefore a dominant presence of mesopores in the microstructure. Otherwise, a type I isotherm associated with monolayer formation was noticed for S5 and S6 samples, which indicate a significant share of micropores. The shape of the adsorption branches at the lowest values of relative pressure for these samples suggest a broader range of the pore size distribution including wider micropores and narrow mesopores. Thus, their isotherms may be precisely identified as type Ib. The hysteresis of the isotherms is generally explained as the effect of capillary condensation, which is often associated with the adsorption metastability. Therefore, the thermodynamic equilibrium is established only during the desorption. This may indicate an open-ended geometry of the pores. 

**Figure 6:**
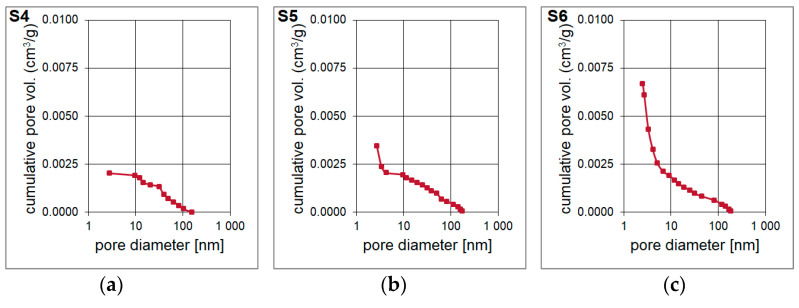
(**a**–**c**) Pore volume of the porous silicon oxycarbide materials, abbreviation: vol.—volume.

**Figure 7:**
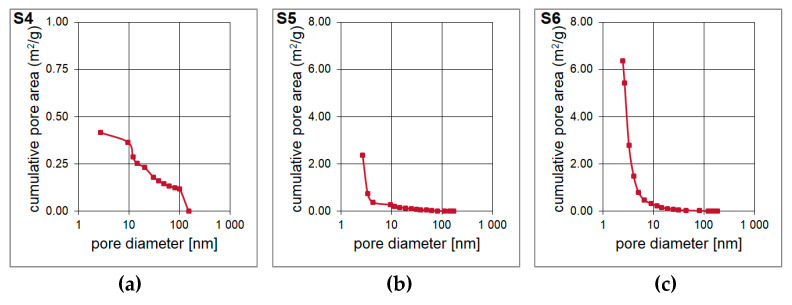
(**a**–**c**) Pore area of the porous silicon oxycarbide materials.

The result of the surface area and pore volume assessment is shown in Table 4. The surface area of the S4–S6 samples was determined based on Single Point method as well as BET and Langmuir models. The surface area of the material is increases with the increase of the molar ratio of T:D structural units in the synthesized polysilsesquioxanes sols. It is probably determined by the cross-linking of the initial xerogel. The higher the T:D ratio, the higher the surface area of the obtained materials. During the pyrolysis, gases from bond redistributions (Si-C(H) and Si-O) are released. This leads to the formation of channels/capillaries. Most likely, the free carbon phase is also involved. It is important to underline that the results of Single Point method are mainly used in routine measurements whilst Langmuir model, which is based only on the monolayer formation, often overestimates the results. Therefore, the data obtained with the application of BET model should be the most accurate. The BET surface area for S6 sample was the highest, reaching 268.49 m^2^/g. T-Plot method let to assess the micropore area and volume. Both parameters were the lowest for S4 sample and significantly higher for S5 and S6 samples. They were the highest and equal to 236.67 m^2^/g and 0.1099 cm^3^/g, respectively, for the latter one. Pore volume and pore area distributions (Figure 6 and Figure 7) were determined based on Barrett-Joyner-Halenda (BJH) analysis. They confirm the mesoporous microstructure for S4 sample and the microporous microstructure with the presence of narrow mesopores for S5 and S6 samples. 

## 4. Conclusions

Porous ceramic silicon oxycarbide materials were prepared by the sol-gel method and subsequent pyrolysis at 800 °C. Materials were obtained after thermal processing from all synthesized samples. Structural analysis (XRD, FT-IR) enables us to state that samples before the pyrolysis are characterized with the ladder-like structure, while after the pyrolysis, the amorphous structure characteristic for SiOC materials was detected. The ^29^Si and ^13^C MAS NMR experiments clearly indicate obtaining SiOC as the final product. The analysis shows that [SiO_3_C] (T structural units) is the dominant phase, followed by the [SiO_2_C_2_] (D structural units) phase. BET studies identify mesoporous microstructure for the sample T:D ratio 1:1, whereas microporous microstructure with narrow mesopores is obtained for the samples T:D ratios 2:1 and 4:1, which results in a higher BET surface area. Materials, which are characterized with high surface area (more than 100 m^2^/g), porous structure, thermal stability, and chemical inertness, can be used as catalyst supports. Prepared materials present similar physicochemical properties to aluminum oxide, which made them a perspective material for catalysts carriers. Other potential applications include high-temperature gas sensors, filters, adsorbents, or advanced drug delivery systems.

## Figures and Tables

**Table 1:** Detailed FT-IR spectra analysis for the xerogels (S1–S3 samples), abbreviations explained under the table.

**Band Position (cm^−1^) and Characteristics**			**Type of the Vibration**
**S1**	**S2**	**S3**	
-	3446 br, m	3438 br, m	ν_as_ and ν_s_ O-H in H_2_O
2969 m	2975 m	2973 m	ν_as_ of C-H in Si-CH_3_
2911 w	2914 w	2928 w	ν_s_ of C-H in Si-CH_3_
-	1627 w	1627 w	δ_s_ H-O-H in H_2_O
1412 w	1413 w	1411 w	ν_as_ of C-H in Si-CH_3_
1271 s	1273 s	1272 s	ν_s_ of C-H in Si-CH_3_
-	1123 s	1123 s	ν_as_ of Si-O-Si rings
1090 s	-	-	ν_as_ of Si-O-Si rings
1030 vs	1028 vs	1032 vs	ν_as_ of Si-O-Si bridges
857 m	856 m	854 m	δ of C-H in Si-CH_3_
800 s	798 s	-	ν_as_ of Si-C in Si-CH_3_
-	782 s	782 s	ν_as_ of Si-C in Si-CH_3_
702 w	702 w	701 w	ν_s_ of Si-C in Si-CH_3_
573 w	572 w	572 w	ν_s_ of Si-O-Si rings
439 m	443 m	443 m	δ of O-Si-O

Abbreviations: vs—very strong, s—strong, m—medium, w—weak, br—broad, ν—stretching vibration, δ—bending vibration (subscripts: s—symmetric, as—asymmetric).

**Table 2:** Detailed FT-IR spectra analysis for the silicon oxycarbide materials (S4–S6 samples), abbreviations used as in Table 1.

**Band Position (cm^−1^) and Characteristics**			**Type of the Vibration**
**S4**	**S5**	**S6**	
3436 br, m	3433 br, m	3433 br, m	ν_as_ and ν_s_ O-H in H_2_O
2927	2925	2923	ν_as_ of C-H in Si-CH_3_
2857 w	2854 w	2853 w	ν_s_ of C-H in Si-CH_3_
2257 w	2258 w	2590 w	ν Si-H in O-Si-H
1705 w	1709 w	1708 w	ν C=O in oxidized carbon phase
1630 w	1631 w	1628 w	δ_s_ H-O-H in H_2_O
1361 w	1361 w	1361 w	ν_as_ of C-H in Si-CH_3_
1276 w	1276 w	1276 w	ν_s_ of C-H in Si-CH_3_
1066 vs	1064 vs	1058 vs	ν_as_ of Si-O-Si bridges
876 w	876 w	875 w	δ Si-H in O-Si-H, ν Si-C
807 m	805 m	806 m	ν_s_ of Si-O-Si
453 m	454 m	449 m	δ of O-Si-O

**Table 3:** Calculated percentage share of the structural units in the structure of S4 sample based on ^29^Si MAS NMR experiment.

**Structural Unit Type**	**Chemical Shift (ppm)**	**% Share**
Q^4^ [SiO_4_]	−108.8	4.5
Q^3^ [SiO_3_OH]	−100.7	12.4
T [SiO_3_C]	−64.1	48.7
D [SiO_2_C_2_]	−36.0	5.4
D^H^ [HSiO_3_]	−24.3	16.8
X [SiC_4_]	−11.1	9.3
M [SiOC_3_]	3.0	2.9

**Table 4:** Surface area and pore volume data for samples S4–S6.

**Parameter**	**Sample**		
	**S4**	**S5**	**S6**
Single Point Surface Area (m^2^/g)	175.64	257.16	277.20
BET Surface Area (m^2^/g)	167.41	248.31	268.49
Langmuir Surface Area (m^2^/g)	222.23	327.47	354.73
t-Plot External Surface Area (m^2^/g)	10.05	24.39	31.83
t-Plot Micropore Area (m^2^/g)	157.36	223.92	236.67
t-Plot Micropore Volume (cm^3^/g)	0.0739	0.1040	0.1099

## Data Availability

The data presented in this study are available on request from the corresponding author.
